# Computational Modeling of Skull Bone Structures and Simulation of Skull Fractures Using the YEAHM Head Model

**DOI:** 10.3390/biology9090267

**Published:** 2020-09-04

**Authors:** Alcino Barbosa, Fábio A. O. Fernandes, Ricardo J. Alves de Sousa, Mariusz Ptak, Johannes Wilhelm

**Affiliations:** 1 TEMA: Centre for Mechanical Technology and Automation, Department of Mechanical Engineering, University of Aveiro, Campus de Santiago, 3810-193 Aveiro, Portugal; alcino.rafael@live.ua.pt (A.B.); rsousa@ua.pt (R.J.A.d.S.); 2 Faculty of Mechanical Engineering, Wroclaw University of Science and Technology, Łukasiewicza 7/9, 50-371 Wrocław, Poland; mariusz.ptak@pwr.edu.pl (M.P.); johannes.wilhelm@pwr.edu.pl (J.W.)

**Keywords:** finite element method, skull, trabecular bone, cortical bone, biomechanics, head injury, sutures, skull fractures

## Abstract

The human head is a complex multi-layered structure of hard and soft tissues, governed by complex materials laws and interactions. Computational models of the human head have been developed over the years, reaching high levels of detail, complexity, and precision. However, most of the attention has been devoted to the brain and other intracranial structures. The skull, despite playing a major role in direct head impacts, is often overlooked and simplified. In this work, a new skull model is developed for the authors’ head model, the YEAHM, based on the original outer geometry, but segmenting it with sutures, diploë, and cortical bone, having variable thickness across different head sections and based on medical craniometric data. These structures are modeled with constitutive models that consider the non-linear behavior of skull bones and also the nature of their failure. Several validations are performed, comparing the simulation results with experimental results available in the literature at several levels: (i) local material validation; (ii) blunt trauma from direct impact against stationary skull; (iii) three impacts at different velocities simulating falls; (iv) blunt ballistic temporoparietal head impacts. Accelerations, impact forces, and fracture patterns are used to validate the skull model.

## 1. Introduction

Road traffic accidents are one of the leading causes of mortality in the world, resulting in approximately 1.35 million deaths [1]. Head injuries play a significant role, being also one of the main outcomes of work accidents [2]. Skull fractures may range from a simple nose fracture (one in the Abbreviated Injury Scale, AIS) to a five to six for depressed skull fractures that usually result in a subdural hematoma (SDH), with bones penetrating and tearing veins within the subdural space or, in the worst-case scenario, crushing the cranium.

There are many types of cranial fractures but one major cause: an impact or a blow to the head, strong enough to fracture the bone. The type of fracture depends on the impact force, its pulse, the impacted location, and the shape of the impacting object. Cranial fractures can occur without evidence of brain damage [3]. Nevertheless, the incidence of intracranial hematomas is higher among patients with cranial fractures [4].

The skull is a layered structure comprised of a compact inner and an outer table separated by a porous diplöe layer. When a layered panel is transversely loaded, four types of mechanisms can lead to transverse deflection: membrane deformation, bending deformation, shear deformation, and local core compression and puncture of the skull [5]. The compact bone is a dense calcified tissue that forms the outer layer of all bones and surrounds the cancellous bone. Overall, a bone is neither ductile nor fragile, but a combination of both, presenting a quasi-brittle behavior. The mineral part is more unstable/brittle and the organic part (collagen) is more ductile [6]. The quasi-brittle behavior shows the gradual decrease of stress after reaching the yield stress or elastic limit of the material [7].

Lynnerup et al. [8] reported that neither trabecular skull thickness nor total skull thickness is significantly associated with an individual’s gender, weight, or height. Additionally, a statistically significant correlation between trabecular layer thickness and total skull thickness was found. Lillie et al. [9] reported interesting data regarding the thickness of both cortical and trabecular bones. The reported values of cortical thickness range between 1 and 2.6 mm, varying with age and gender (20 to 99 years old).

The human skull consists of approximately twenty-two bones, mostly connected by ossified joints, the so-called sutures. Sutures are flexible joints that allow the bones to grow evenly as the brain grows and the skull expands. All skull bones are connected by cranial sutures, which are mainly composed of collagen. There are thirteen sutures in the human skull, four of which are considered the main ones: the coronal suture, the sagittal suture, the squamous suture, and the lambdoid suture. The literature lacks a consensus on the mechanical properties of cranial sutures. This is due to their different spatial distribution of collagen fibers, which results in different geometries and mechanical properties [10]. These properties vary significantly with age, for instance, the Young modulus of cranial sutures is reported to go from 50 to 200 MPa in infants [11,12,13] and 4 GPa in adults [14].

To better understand traumatic head injury, mechanical and mathematical models of the human head have been developed. Essential to the development of these models is the study of the mechanical behavior of the materials and structures which constitute the human head. The main building component of the bone tissue is mineralized collagen fibril (ossein) [10]. The collagen fibers at the outer and inner tables are arranged hierarchically. On the contrary, in the trabecular bone, collagen fibrils are oriented irregularly, and the structure is spongy. The position of fibers in the bone affects its mechanical properties.

Many postmortem investigations have been conducted using human and animal heads, and also physical head and in vitro models. These experiments provided valuable data, which combined with the development of computational techniques, resulted in the development of numerical head models, mainly resorting to the finite element method (FEM) [4]. Although consisting of a numerical procedure to determine approximate solutions for physical or mathematical problems, it requires a precise representation of complex geometries, boundary conditions, and suitable constitutive laws. These models have been used in several fields, from the study of sports trauma to traffic accident reconstruction and forensic research [15,16,17,18,19]. FE models of the skull have also been used in the study of cranioplasty implant systems for the human skull [20,21].

One of the first attempts to model the behavior of the human head response through the FEM was made by Hardy and Marcal [22], who developed a 2D FE model of the skull, later enhanced by Shugar [23], who added an elastic brain firmly attached to the skull. Since then, many FEHMs have been developed, evolving towards more complex models constituted by more intracranial features and modeled with complex non-linear constitutive laws [24,25]. However, most of the attention has been devoted to the brain and other intracranial structures. The skull, despite playing a major role in direct head impacts, is often overlooked and simplified. Usually, at least one of the following simplificative assumptions can be found in the literature: constantly thick skulls, homogeneous skull material, linear elastic mechanical response, and sometimes a lack of validation against experiments regarding skull mechanical response under loading neither its fracture propagation. In the present work, the authors try to enhance the state-of-art by covering all of these points, including variable thicknesses, inclusion of sutures, and segmentation of compact bone and diplöe (all modeled with hexahedral elements), damage models for crushing of trabecular bone and fracture propagation, and finally, a set of distinct validations from specific crushing of trabecular bone to depressed skull fractures.

In the literature, there are a few studies where cadaveric experiments were simulated in order to validate the model, focusing on the skull response during dynamic experiments. For instance, Sahoo et al. [26] simulated the drop tests performed by Yoganandan et al. [27] aiming to validate the skull response under different impact velocities, representing different and possible impact energies in real-world falls. Later, Asgharpour et al. [28] used the same FEHM and simulated the frontal impact tests performed with cadaver heads in a two-pendulum apparatus by Verschueren et al. [29]. In another example, Cai et al. [30] developed a FEHM to simulate the skull fracture experiment from Yoganandan et al. [31], in which the skull is impacted by a hemispherical anvil, and also the lateral head drop experiments from Yoganandan et al. [27] and finally, the blunt ballistic temporoparietal impact experiment from Raymond et al. [32]. However, Cai et al. [30] modeled all the cranial structures as linear elastic materials, which negatively affect the model response, especially for the simulation and prediction of skull fractures.

Other skull models have been developed for particular studies, studying very specific subjects, different from the works mentioned above that comprise the entire head structure. Chamrad et al. [33] reviewed different ways to model the human skull and concluded that the skull model must have both types of bone. Additionally, the case of cortical bone modeled with shell element-layers filled with solid elements that represent trabecular bone tissue, it was found to be less precise and feasible than modeling it with solid elements. Chamrad et al. [33] also compared different cortical thicknesses, 1 and 2 mm, and better results were achieved with the thinner cortical layers. Nevertheless, the response might have been influenced by the simply linear-elastic material models employed.

In another study, Ptak et al. [10] investigated how dynamic loading affects the mechanics of cranial sutures and surrounding bones and concluded that the stiffness of cranial sutures plays an important role in stress distribution and energy absorption. Thus, it is possible to conclude that the modeling of cranial sutures is vital for realistic modeling and simulation of skull fractures. Nevertheless, this model was neither assembled nor validated and the intracranial components were missing. Recently, an advanced FE model of the human skull for a child was developed by Wilhelm et al. [34]. Nevertheless, the particular validation for the skull was not carried out due to the lack of experimental data for the validation of the child head models. A similar methodology was adopted in this study, re-modeling the skull of the YEt Another Head Model (YEAHM), which can be compared with experimental data available in the literature for adult subjects.

This work introduces a new skull model to the YEAHM, substituting the linear elastic homogeneous skull developed in Fernandes et al. [35]. Since this model does not differentiate the types of skull bone, modeling it as a linear-elastic homogenous solid, in the present work, a new skull model is developed for the YEAHM. It is based on the original geometry and focuses on the differentiation and segmentation of compact and cancellous tissue and the inclusion of cranial sutures, having variable thickness across different sections and based on craniometric data.

These structures are modeled with constitutive models that consider the non-linear behavior of skull bones and also the nature of their failure. Several validations are performed, comparing the simulation results with experimental results available in the literature at three levels: (i) local material validation [36]; (ii) blunt trauma from direct impact against a stationary skull [31]; (iii) three impacts at different velocities simulating falls [27]; (iv) blunt ballistic temporoparietal head impacts [37]. Accelerations, impact forces, and fracture pattern results are compared with experimental data from the literature in an attempt to validate the skull model.

## 2. Materials and Methods

**Figure 1:**
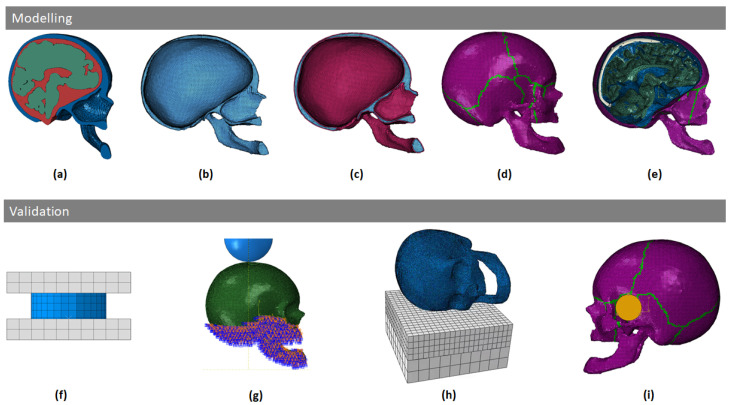
Modeling and validation stages: (**a**) YEAHM original skull model; (**b**) trabecular bone structure; (**c**) trabecular bone covered by the cortical layers; (**d**) sutures inclusion; (**e**) adding of intracranial contents; (**f**) trabecular bone compression; (**g**) blunt impact against stationary skull; (**h**) simulation of falls—lateral impacts; (**i**) blunt ballistic temporo-parietal head impacts.

The YEAHM, as originally developed by Fernandes et al. [35], was constituted by the skull, brain, and cerebrospinal fluid (CSF), was further enhanced by Migueis et al. [38] adding the bridging veins and the transverse and superior sagittal sinuses and by Costa et al. [39] pressurizing these hollow structures and validating them against experimental studies from Monea et al. [40] and Depreitere et al. [41], in order to accurately capture the onset of bridging veins rupture and SDH prediction. The model was also previously validated against the experiments of Nahum et al. [42] and Hardy et al. [35,43,44]. The latter model is employed as the base model in the present study. Since the development and validation of YEAHM can be found in the literature, only data referring to the skull model here developed will be addressed. Figure 1 presents the major modeling and validation stages, which will be thoroughly addressed in the following sections.

### 2.1. Skull Modeling

The first step in the development was to segment the cranial bones. If properly carried out, cortical and trabecular bones will have distinct geometries and material properties. As referred, cranial sutures were also modeled.

Linear hexahedral finite elements are the selected choice, instead of the previously employed quadratic (second-order) tetrahedral elements. Tetrahedral finite elements are easier to mesh considering geometrical irregularities, but first-order ones are excessively stiff, leading to numerical pathologies such as volumetric locking as reported by Fernandes et al. [35] for the nearly incompressible brain matter. On the other hand, second-order tetrahedral elements are extremely CPU intensive. Linear hexahedral elements have the advantage of having fewer nodes while giving as accurate results as second-order tetras, reducing the computation cost [45]. Nevertheless, when modeling complex geometries with linear hexahedral elements, it is necessary to address their sensibility to the corner angle.

Since the skull is a layered structure, shell elements could be an option in some FEHMs to model the skull [26]. However, in this work, there are mainly three reasons for the use of solid elements instead of shells for the cortical bone:

1. Construction of the model: with solid finite elements it is possible to quickly make geometrical adjustments to the model and adapt zones more easily than in the case of shells. In addition, since the head is also a layered structure itself (meninges, skull, scalp, etc.), solid elements make it easier to model additional layers in future works.
2. Solids also have the advantages of handling double contact better and have a more accurate description of the stress gradient over the thickness, contrary to shells based on plane stress assumptions. Full 3D material laws can be employed without simplifying assumptions like in the case of shells.
3. Solid elements are more appropriate than shell elements to study the fracture phenomena of the skull.

#### 2.1.1. Geometrical Modeling

The YEAHM skull geometry is based on computer tomography (CT) scans, more specifically 460 slices at 1.5 mm intervals images. The geometry generated in Fernandes et al. [35] was used as the base in this study. Meshmixer (Autodesk, San Rafael, CA, USA) was employed to obtain one internal surface and one external surface of the skull. The original skull model was then separated into two distinct parts, defining the cut in the foramen magnum, a large opening through the occipital bone located in the center of the posterior fossa of the neurocranium.

Following, an inward thickness of 1.5 mm was attributed to each part based on the range of thicknesses reported for the cortical layer (1 and 2 mm) [9], to be used as a virtual reference border between the two bone constituents. The geometry was then imported into SolidWorks (Dassault Systèmes, Vélizy-Villacoublay, France) to create a solid part. The FE mesh of the solid trabecular component was created with the mesh generator algorithms from HyperMesh (Altair, Troy, MI, USA).

The mesh generation was controlled by setting two parameters, the desired element size of 2 mm and a minimum Jacobian determinant of 0.3 as parameters, to ensure low-distortion, good aspect ratio hexahedral elements. Those specific parameters were defined to ameliorate convergence and diminish the overall CPU cost on further simulations.

**Figure 2:**
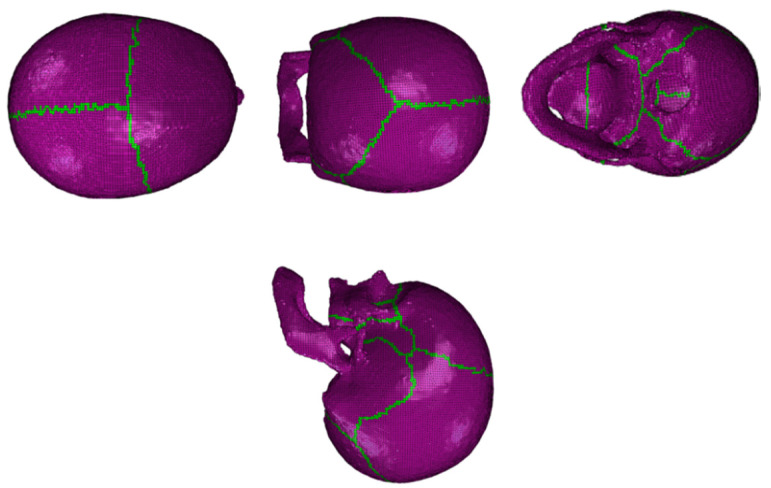
Visualization of the final complete model in Abaqus with trabecular bone, cortical bone (1.5 mm), and cranial sutures.

The cortical layer was created based on the trabecular mesh by extruding a 1.5 mm layer of hexahedral elements sharing nodes with the trabecular core. Overall, the mesh element quality is good and faithful to the geometry. Lastly, the two components were segmented to create a component of cranial sutures, using reference images from a 3D atlas. The skull hexahedral mesh is then imported into the Abaqus solver to run the simulations, bearing in mind that the final part would be sectioned with the three different components (trabecular, cortical, and cranial sutures). The final model (Figure 2) is constituted by these distinct components: a trabecular component with 92,300 hexahedral elements, a cortical component with 133,045 hexahedral elements, and a cranial sutures component with 12,271 hexahedral elements. In addition, the interfaces between the components have shared nodes.

After creating the skull model, the intracranial components (namely the brain, the CSF, the BVs, and the SSS) from the existing YEAHM model were assembled to the new skull model in Abaqus (Figure 1e). For this purpose, during the modeling process of the skull, the coordinate system and its origin were preserved. Overall, the model contains 1,202,015 elements, of which 20% (237,616) form the skull.

#### 2.1.2. Material Modeling

**Figure 3:**
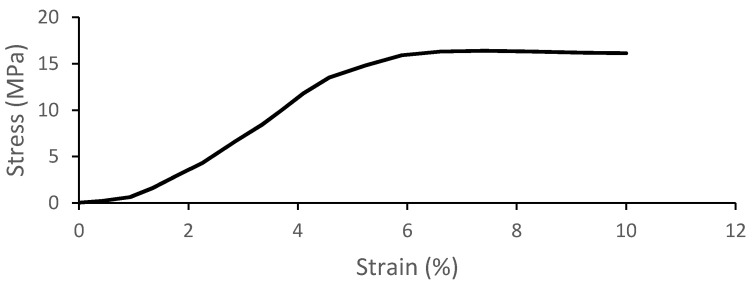
Stress–strain behavior of trabecular bone under compression loading [36].

The geometries of the cortical and trabecular bones along with the cranial sutures were modeled resorting to material models available at the Abaqus material library. The trabecular or spongy bone was modeled as an elastic-plastic material since, from all the bones, this type behaves more like, as the name indicates, a sponge between the cortical bone tables and without exhibiting an S-shaped stress–strain curve for higher deformations, typical of crushable foams. This model is also the most used one on the FEHMs available in the literature [24]. The compression tests performed by Boruah et al. [36] were used to fit the model in Abaqus. Figure 3 shows the stress-strain curve obtained by Boruah et al. [36] and used in this work to characterize the material behavior. Moreover, the material was considered isotropic with a density of 1500 kg/m^3^, a Poisson’s ratio of 0.05, and a Young modulus of 1000 MPa, values found throughout the literature [46].

Based on the information found in the literature regarding the cortical bone and sutures, knowing that these types of tissues present a quasi-brittle mechanical behavior, a material model capable of simulating brittle fracture is necessary. Abaqus provides such a material law to model the brittle behavior of materials such as ceramics, concrete, and even glass [47]. The removal of elements based on a brittle failure criterion is also employed. The mechanical behavior described by this model is driven by two stages: prior to and after cracking. Prior to cracking, a linear elastic isotropic material model defines its mechanical behavior, as described by the classical Hooke’s Law. The elastic behavior values chosen for both cortical bone and cranial sutures can be seen in Table 1.

**Figure 4:**
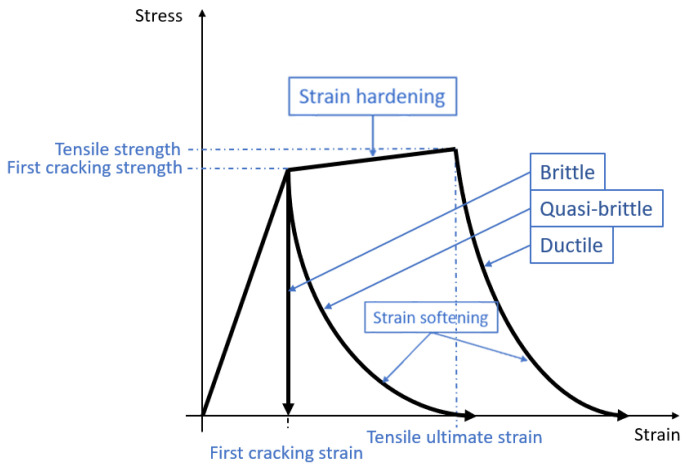
Cracking modeling—strain softening and strain hardening under uniaxial tensile loading.

**Figure 5:**
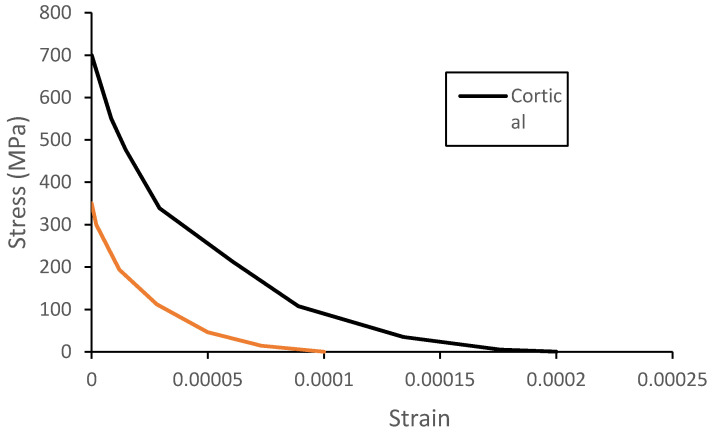
Post-failure stress–strain curve for both cortical and sutures tissues.

To define crack initiation and the behavior of bone tissue after cracking, three modules must be defined: a post-failure stress–strain relation, a shear retention model, and a brittle failure criterion (Figure 4). The post-failure stress–strain relation defines the post-failure stress, σ*_t_^I^*, as a function of the strain across the crack, ε^ck^_nn_, modeling it after its initiation. Figure 5 shows the values used for both tissues.

**Figure 6:**
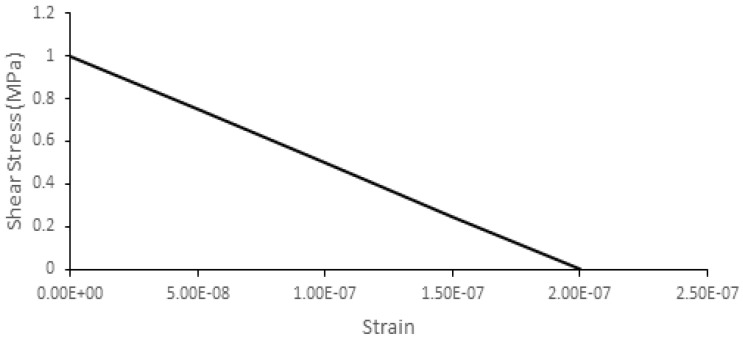
Shear retention model values for both cortical and sutures tissues.

Regarding the shear retention model, it requires the definition of the post-cracked shear stiffness as a function of the opening strain across the crack. This relation is defined by:

(1) $$G_{c}=\omega (\varepsilon_{nn}^{\mathrm{ck}})G^{\;}$$

where $\varepsilon_{nn}^{\mathrm{ck}}$ is the strain after cracking, ω is the shear retention factor and *G_c_* is the cracked shear modulus. The last one reduces as the crack opens. Figure 6 shows the relation employed for the specific case of both cortical and sutures tissues.

The material cracking is defined by an often-used criterion to predict the failure of brittle materials, the Rankine criterion, which is based on the maximum normal stress and determines the crack initiation, deleting the corresponding element. The brittle failure criterion allows the definition of the number of local direct cracking strain components (maximum three) at a material point and the failure strain to cause element failure. When the material point fails, all the stress components are set to zero. If all the material points in an element fail, the element is removed from the mesh. The number of material points required for element failure can be defined. In this case, for the cortical bone, one material point was set as the requirement for element failure with a direct cracking failure strain of 0.0006. Similarly, for the cranial sutures, one material point was set as the requirement for element failure but with a reduced direct cracking failure strain of 0.0004.

### 2.2. Skull Model Validation

The selection of the experimental studies to perform the validations is based on commonly used tests in the literature and the necessity for specific validations such as the trabecular bone [36]. After validating the compression of this type of bone, simulations can be performed covering the entire skull eliminating the diplöe as a variable. Additionally, in the literature, no study was found addressing experimental testing solely on sutures without the influence of cortical tissue, and vice-versa. Therefore, after trabecular bone validation, the skull model was validated by simulating head blunt trauma and head impacts resulting from falls [27,31]. Additionally, to validate the behavior of the damage/failure models, another experimental study needed to be simulated in order to compare the fracture patterns [37].

Given the kinematics of the analyzed problems, all the simulations performed used the dynamic explicit solver. No mass scaling technique was employed. The interaction between the parts was considered as frictionless general contact, in order to reduce CPU cost and also given the lack of information on the friction coefficients for most of the experiments. Additionally, the majority of the contacts happened against metallic components with low roughness and in a linear fashion, with minimal tangential displacements.

#### 2.2.1. Trabecular Bone Compression—Local Material Validation

For the trabecular bone validation, the uniaxial compression tests conducted by Boruah et al. [36] were simulated. Boruah et al. [36] tested a total of ten adult male post-mortem human surrogates, representing the 50th percentile adult male with an upper age limit of 70 years. A skull CT was used to identify ten anatomical locations on the right half of the calvarium for harvesting cores with the locations chosen to avoid sutures.

The scalp was removed, and cylindrical-shaped samples were harvested on the right side of the calvarium. The process was guided by a clinical CT to precisely remove the samples for each human surrogate on the desired locations and avoid specific anomalies such as deformation and table curvature. A total of 84 cores with a diameter of 18.24 mm were obtained from the right calvaria of the ten subjects. Table 2 indicates the mean thickness of each layer and the corresponding standard deviation. The samples were potted in a minimal amount of polyester resin to provide two flat and parallel surfaces for mounting the specimen on the uniaxial test rig. 

To simulate the compression of trabecular bone and to validate the constitutive strategy and material properties employed for trabecular bone, a cylindrical sample was developed based on the average measurements for outer and inner tables and trabeculae, as obtained in Boruah et al. [36], reducing the probability of errors due to geometrical deviations.

For this reason, for numerical simulation purposes, the mean dimensions presented in Table 2 were adopted. The compressibility test setup consisted of the core specimens being loaded with a ramp displacement applied to the outer table at a target rate of 15 mm/s, with the outer table facing the actuator. Figure 1f shows the numerical setup, which consists of two rigid parts, the bottom one fully constrained, and the top one with one degree of freedom. The velocity of 15 mm/s was prescribed to the latter. The cylindrical samples were modeled with hexahedral elements, analyzing the element size, the type of integration, and the influence of the damage model.

#### 2.2.2. Skull Vertex Impact Experiment—Blunt Trauma from Direct Impact against a Stationary Skull

In Yoganandan et al. [31], 21 specimens were loaded at quasi-static and dynamic rates. From these tests, a specific test, specimen number 7, was chosen to be recreated on a simulation. This particular test consisted of a dynamic rate test where a hemispherical anvil with 96 mm of diameter and a mass of 1.213 kg impacted on the vertex of the cranium (on the sagittal sutures) at 7.2 m/s. This one was chosen because the impact location contained for the most part sutures and also because force–deflection curves were provided in Yoganandan et al. [31]. Additionally, the specimen was from a 65-year-old man, having similar conditions to the specimen from whose medical images were used to create YEAHM, e.g., the craniometric data [35].

The specimens were prepared with a fixation device designed to achieve rigid boundary conditions at the distal end. Therefore, the skull model was properly constrained to represent the experimental boundary conditions. The bottom of the model was fully constrained as in the experiments [31]. Figure 1g presents the numerical setup, showing the constrained area of the skull and the rigid hemispherical impactor. In this simulation, an initial velocity of 7.2 m/s was set to a 1.213 kg rigid hemispherical anvil with a diameter of 96 mm and with an initial speed of 7.2 m/s, which impacted against the vertex of the skull.

#### 2.2.3. Lateral Head Impact Experiment—Three Impacts at Different Velocities Simulating Falls

In Yoganandan et al. [27], post-mortem human subjects were subjected to impacts on the lateral side of the cranium. Three linear accelerometers at each site/region were rigidly fixed onto a metal cube that was then rigidly mounted to a contoured plate. This plate was attached to the cranium using screws at each corner.

The specimens were impacted with successively increasing input energies until fracture. The stopping criterion was a decrease in force occurring with increasing impact velocity, or the impact force being closer to the rated limit of the load cell. A 40-durometer padding (50 mm thickness) material served as a stationary object onto which the head impacted. The dynamic loading was accomplished using free-fall (drop) techniques where the specimen was completely unconstrained. 

The FEHM can be validated by comparing the accelerations at the three regions where accelerometers were placed in the experiment. The resultant accelerations from the experiments were filtered at 1000 Hz with the SAE recommended filtered. The same protocol is employed to filter the numerical results.

**Figure 7:**
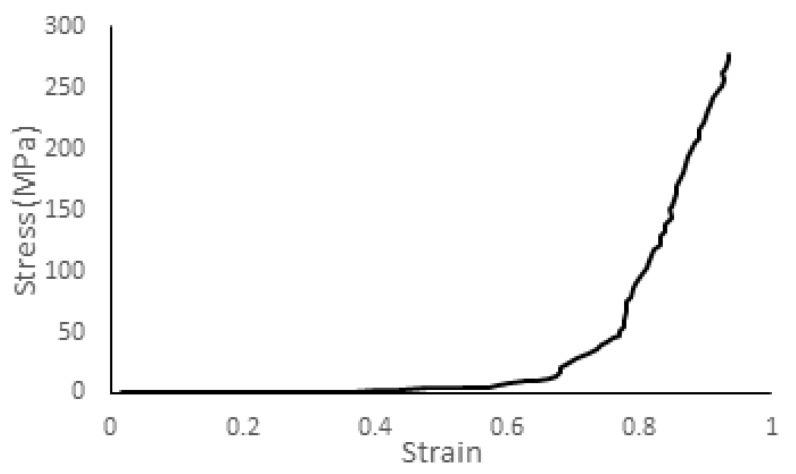
Stress–strain curve of the neoprene from uniaxial compression tests [49].

Figure 1h presents the numerical setup, where the head model was positioned laterally to the surface of the pad. Velocities of 6, 4.9, and 3.5 m/s were given to the head so that it would impact against a 40-durometer neoprene padding (50 mm thickness) material laying on a rigid structure [27,48]. Yoganandan et al. [27] did not give any reference to the material nor its mechanical properties, only stating that it was a 40-durometer layer. Therefore, data from ElGawady et al. [49] and Sahoo et al. [26] were used to model the pad. From ElGawady et al. [49], compression data from the tests performed on a 40 durometer neoprene pad, and in the second [26], the density and Poisson’s ratio used in Yoganandan et al. [27] were revealed. The pad was modeled as a Hyperfoam material with a density of 4320 kg/mm^3^, a Poisson’s ratio of 0.43, and the uniaxial compression test curve is shown in Figure 7.

#### 2.2.4. Blunt Ballistic Temporo-Parietal Head Impacts

Huang et al. [37] performed two blunt ballistic temporoparietal head impacts carried out on a post-mortem human subject. A rigid, flat-faced 38.1 mm diameter projectile with a mass of 0.1 kg was used for all impacts. The impactor was aligned so the contact face struck the specimen normal to the skin surface. Two impact conditions were performed, one to each of the two bilateral temporoparietal regions. Condition A was performed at a target velocity of 20 m/s to the right side while condition B was targeted for an impact velocity of 35 m/s to the left side. 

To validate the model, both conditions were simulated, especially condition B since fracture patterns were provided for this case. This experiment provides data from temporoparietal head impacts of post-mortem human subjects necessary to validate FE models as a prediction tool for localized depressed skull fractures. 

For this simulation, a 0.1 kg cylindrical impactor with 38.1 mm of diameter was modeled. Two impacts were performed, one at 35 m/s and another at 20 m/s. The anvil impacts the head on the temporoparietal region, with a targeted impact point (25 mm anterior to the external acoustic meatus and 35 mm superior to the Frankfurt Plane). Similar to the experiment, the head model was free to move without restrictions. Figure 1i depicts the numerical setup, with a rigid projectile positioned on the temporoparietal region, following the experiments.

## 3. Results

### 3.1. Trabecular Bone Compression—Local Material Validation

**Figure 8:**
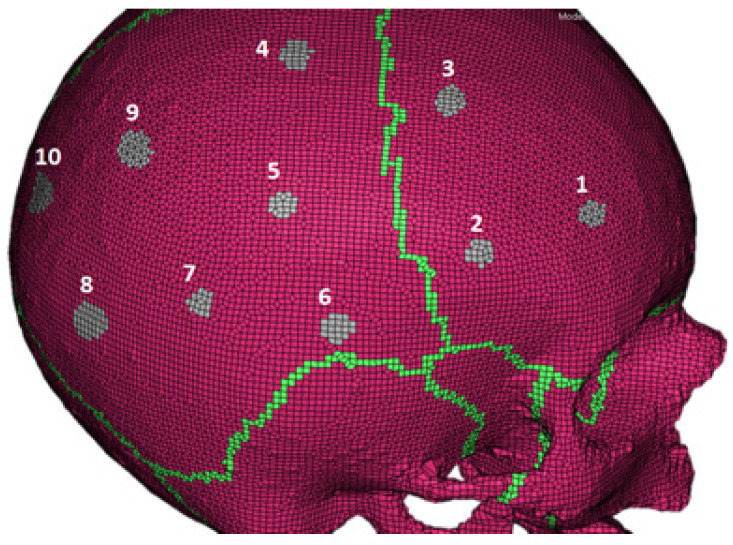
Locations of the samples numbered 1–10 and highlighted in grey. Measurements were carried to find the closest thickness between the numerical model and the samples.

Similarly to the experiment, measurements of the skull numerical model, in what would be the same skull locations as in the experiment, were made to find out what parts of the skull have a higher degree of geometrical similarity with the computational model. Figure 8 shows the locations in the YEAHM skull. Locations 1, 4, and 7 had the best fit regarding the samples’ average thickness of 6.19 mm, as can be seen in Table 3. Therefore, the stress–strain curves obtained for the three samples were the ones primarily used for the model validation.

**Figure 9:**
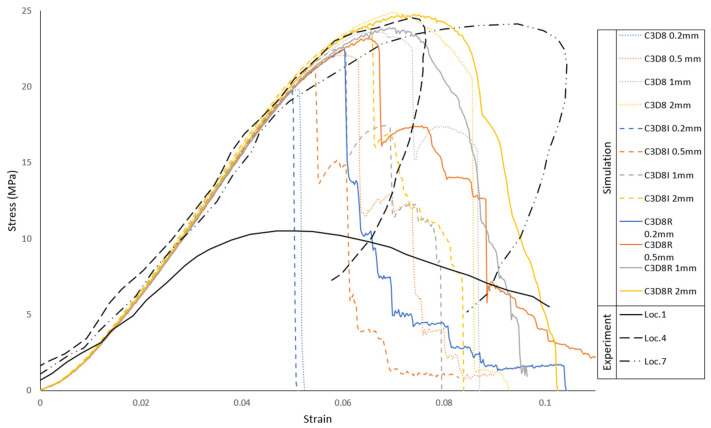
Element type and mesh density results in comparison with the experiments for three locations.

A finite element formulation comparison was made to find the best compromise between accuracy and efficiency. Linear hexahedral elements from Abaqus finite element library with reduced integration and hourglass stabilization (C3D8R), full integration (C3D8), and incompatible modes (C3D8I) were compared. For each type of element, a mesh density analysis was performed with element sizes of 0.2, 0.5, 1, and 2 mm. This mesh sensitivity analysis makes it possible to ensure that the average element size for the trabeculae in the skull model is in good fit with the experiments. It was found that the C3D8R prevailed with the best compromise. Stress–strain curves were obtained to compare the results between simulations and experiments. These results can be seen, altogether, in Figure 9.

**Figure 10:**
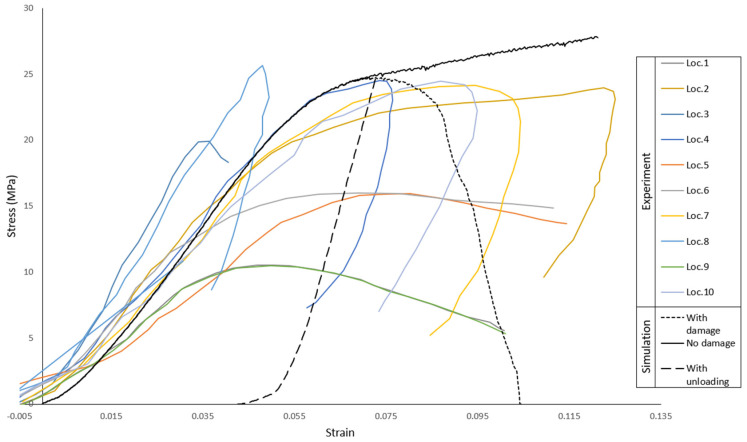
Damage model influence, with and without unloading in comparison with the experiments from all the locations.

Another analysis was made to analyze the inclusion of a damage model, by comparing the trabeculae material with damage, without damage, and with unloading and damage for the 2 mm C3D8R case. The material properties are the ones referred previously coupled with a damage model. For that, a ductile damage model was used with a fracture strain of 0.1, a strain rate of 1.17 s^−1^ [36], and a displacement at failure of 0.1. Both fracture strain and displacement at failure were found through iteration. The results of this comparison are plotted in Figure 10. In conclusion, the compression behavior of the trabeculae is deemed validated, and the material model with the C3D8R elements without damage will be the one used in all the remaining simulations.

### 3.2. Skull Vertex Impact Experiment—Blunt Trauma from Direct Impact against Stationary Skull

**Figure 11:**
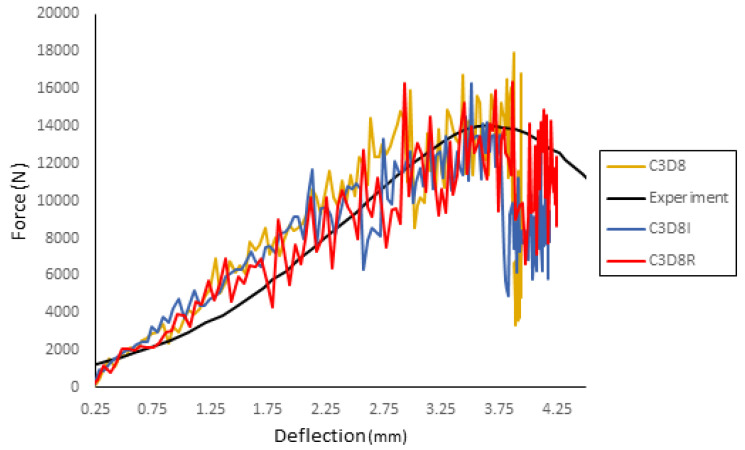
Comparison between element types for the cortical bone and sutures.

**Figure 12:**
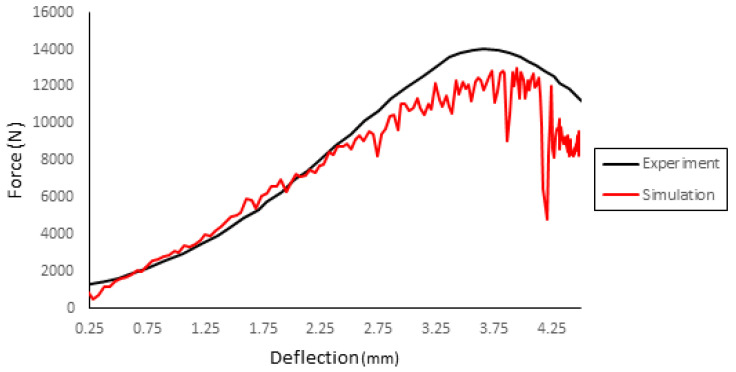
Results of the simulation compared with the experiment results.

A preliminary analysis was made to determine the proper element type for the cortical bone and the sutures. Figure 11 compares these simulations with the experiment. Overall, there is not a significant difference between the different types, concluding that the C3D8R would best suit the model since its behavior is in better accordance with the maximum force found in the experiments. Figure 12 presents the results (force–deflection curve) of the simulation compared with the experiment.

### 3.3. Lateral Head Impact Experiment—Three Impacts at Different Velocities Simulating Falls

**Figure 13:**
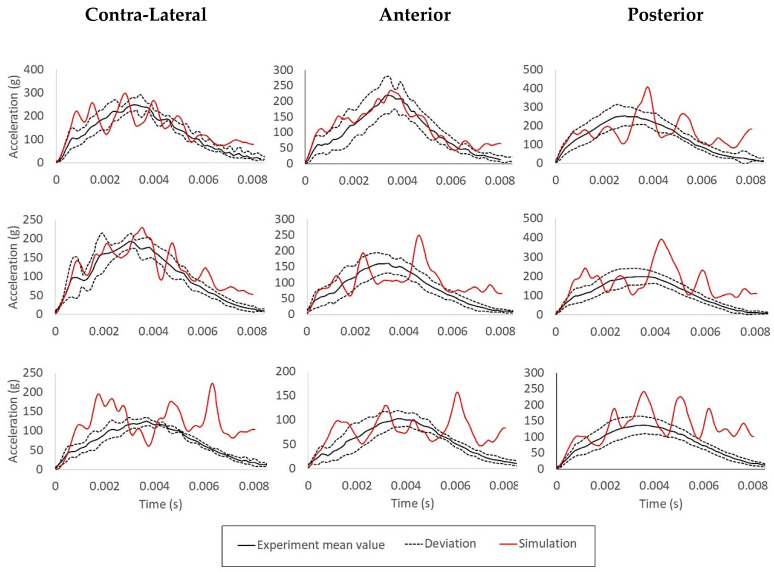
Comparison between the experimental acceleration response expressed as mean ± one standard deviation and the results from the simulations for all the nine cases: impact velocities: 6 m/s, 4.9 m/s, and 3.5 m/s; location: anterior, posterior, and contra-lateral).

Comparisons between results will be done using the acceleration history of three different locations, by taking the average result of 10 nodes from each location (anterior, posterior, and contra-lateral). They are depicted in Figure 13, where the black continuous curves represent the averaged acceleration values, the dashed curves represent the standard deviation from the mean results and the red curves represent the results from the simulations. 

The agreement tends to be much better for impacts with higher velocities but deteriorates for lower impact energies. A plausible reason might be related to the pad material model since there are some uncertainties regarding the material used in the experiments. For instance, this material might be strain rate dependent, and the mechanical properties are based in the literature, without strain rate dependency, which can possibly be a match for 6 m/s impact, but not the case of the lowest impact energy. Since there is not enough information in the literature regarding this point, it will be assumed that the material model of the pad is the major factor for the discrepancies between the simulations and the experiment results.

Additionally, in terms of impact location, the posterior one showed the higher deviations. One possible cause of the deviations for the posterior cases might be the existence of significant differences between the craniometry of the specimens and the YEAHM skull. However, it is not possible to confirm it or refuse this hypothesis since Yoganandan et al. [27] only reported one craniometry measurement, the head breadth. YEAHM’s skull has a head breadth of 145 mm, which is only higher than two of the ten specimens of Yoganandan et al. [27], where the lowest and the largest head breadths are 140 and 178 mm, respectively.

Typically, oscillations such as the ones observed in the simulations results of Figure 13, might reflect numerical instabilities, coming from the contact algorithm or even from the complex material laws employed. However, a deeper investigation allowed the authors to conclude that the acceleration on the chosen nodes (10 nodes, which acceleration runs were averaged eventually) can be amplified by stress-waves on the impacted skull. In the experiment, the accelerometers always have some inertia, unlike in a numerical approach where the nodes have only the material distributed mass. Moreover, the presumed filtering in the experiment resulted in smoother acceleration runs than in FE simulations. Additionally, in the explicit analyses, no mass-scaling techniques were employed. Nevertheless, the use of mass-scaling would ease the instabilities observed. Overall, the extracted curves are still valuable outputs that can be compared against the experiments, with the trend behavior clearly observed. 

### 3.4. Blunt Ballistic Temporo-Parietal Head Impacts

**Figure 14:**
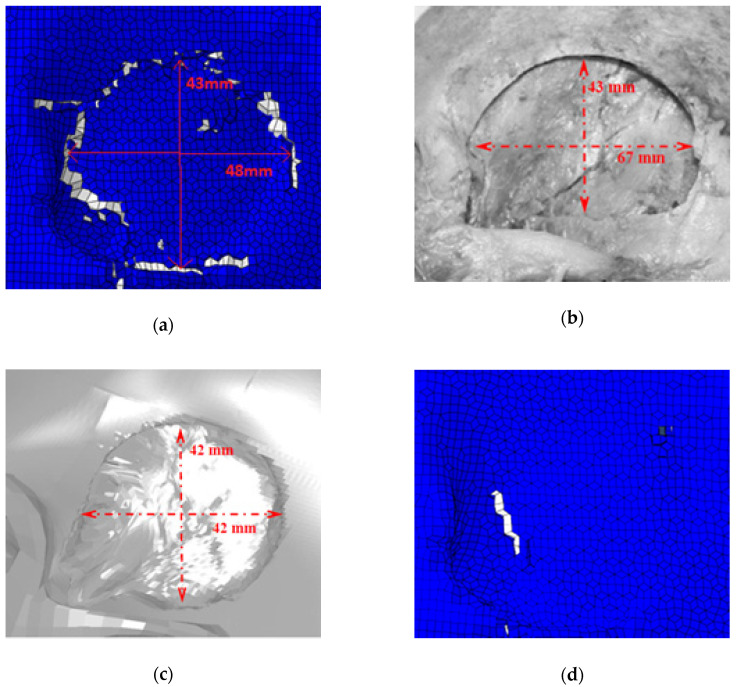
Comparisons of depressed skull fracture pattern obtained with YEAHM and in both Huang’s experiment and simulation: (**a**) fracture pattern from the 35 m/s simulation; (**b**) fracture pattern from the 35 m/s experiment (adapted from [37]); (**c**) fracture pattern from the 35 m/s simulation with the model from Huang et al. [37] (adapted from [37]); (**d**) fracture pattern from the 20 m/s simulation.

In the experiments, the impact at 35 m/s caused a circular depressed fracture, contrary to the 20 m/s impact. Damage to the bone was only observed in the higher energy impact. Figure 14 compares YEAHM’s fracture pattern with both experimental and numerical results obtained by Huang et al. [37]. As can be seen, the results obtained using the YEAHM model updated with the new skull was able to predict a similar depressed fracture to Huang’s study, with a dimension of 43 mm vertically and 48 mm horizontally. The results with this model were closer to the experiments than the numerical model of Huang et al. [37], considering the two reported dimensions, fitting perfectly one of the dimensions of the depressed fractures, and achieving another closer to the experiment. However, in the latter (horizontally), the difference was still significant.

In the experiments carried out by Huang et al. [37], the impactor face struck the specimen normal to the skin surface. This might be one possible cause of the discrepancy in the horizontal measurement. Since the model does not have skin, it is much harder to position the impactor accurately once the reference in the model is the skull surface. Additionally, in the lower energy impact, a tiny fracture was predicted. A possible reason may be also related to the absence of the scalp, so the impact is more concentrated on those specific locations, and the first structure to absorb the impact energy and physically contact a striking object is the skull. This reason is very relevant since the study of Trotta et al. [50] showed that scalp tissue affects head impact biomechanics in a significant way, reducing the linear/rotational acceleration and in some cases causing an important change in the impact kinematics.

## 4. Conclusions

Apart from a few deviations that can be linked to subjects’ variability and uncertain material properties from some experiments, one can state that the validations were performed with success. First, the compression of trabecular bone was validated, achieving a good agreement between numerical and experimental results. The entire skull structure response was validated thanks to blunt impacts with an anvil striking the head and another test method, where the head falls onto the covered ground. These are important to validate the remaining structures of the skull. A good agreement was found between YEAHM’s response and the experimental data. Nevertheless, in the simulation of the experiments resembling falls, significant deviations were found between numerical results and the experiments. The main justification for this deviation is the covering layer of the stationary anvil. The author of the study does not provide the material properties of it, nor a reference, which makes it necessary to assume some of the properties, with some being based on other validation studies from the literature. Nevertheless, the 6 and 4.9 m/s impacts were well simulated, where the main difference is in the lower energy impact, which makes it possible to question the strain rate dependency of the material.

Additional validation was performed to confirm the model’s ability to predict depressed skull fractures. The experiment from Huang et al. [37] was reproduced, and a similar fracture pattern was observed. A better prediction was achieved than the model developed by Huang et al. [37]. Although a better prediction was achieved, there was still a significant deviation in one of the dimensions, and a small fracture was predicted in a non-fracture case. The main justification for this issue is the absence of a scalp structure in the model. Recently, Trotta et al. [50] showed how scalp tissue affects the head impact response. 

The research work carried out presents an update in the YEAHM model, focusing on the enhancement and development of a new skull model, a part usually overlooked and simplified in finite element modeling of the human head. Although most of the attention has been devoted to the brain and other intracranial structures, the authors consider the accurate skull modeling of significant importance as well as other structures, such as the scalp in Trotta et al. [50], which will be addressed in future research works. In this sense, the YEAHM head model can now be employed in a wider range of injury assessments, conjugating the previous developments in terms of SDH predictions with the new capacity to predict skull fractures.

The model developed in this study can be used to optimize headgear (e.g., helmets, headbands, etc.) based on the model response and injury prediction based on reported head injury criteria. The model is also an excellent support tool in the design of products or components where there is a high chance of direct head impact. It can also be a useful tool in forensics science, for instance, in the reconstruction of road accidents or assaults involving head trauma as an outcome from blunt impacts on the head. This model makes it also possible to study some of the deformation mechanisms of the skull and to understand how certain parameters influence its response under an impact. Overall, once properly validated, such a tool allows the evaluation of injuries that may be a possible outcome from head impact scenarios.

## Figures and Tables

**Table 1:** Cortical bone and sutures elastic material properties.

Tissue Type	Density (kg/m^3^)	Young Modulus (MPa)	Poisson’s Ratio
Cortical	1900	20,000	0.21
Sutures	2100	15,000	0.3

**Table 2:** Micro CT measurements [36].

Bone Structure	Thickness (mm)	Standard Deviation (mm)
Outer Table	0.76	±0.29
Inner Table	0.35	±0.15
Trabeculae	5.08	±2.01

**Table 3:** Model thickness measurements in different skull locations.

Location	1	2	3	4	5	6	7	8	9	10
**Skull Thickness (mm)**	6.1	7.1	5.7	6.3	7.3	7.8	6.3	7.3	6.9	5
**Deviation from the Average (mm)**	0.1	0.9	0.5	0.1	1.1	1.6	0.1	1.1	0.7	1.2
